# Application of the “water flow decision pattern” to Asian disaster response

**DOI:** 10.1007/s13201-022-01636-0

**Published:** 2022-04-04

**Authors:** Kyoo-Man Ha

**Affiliations:** Department of Emergency Management, Inje University, 197 Inje-ro, Gimhae city, Gyeongnam 50834 Korea

**Keywords:** Decision pattern, Disaster management, Bounded rationality, Failure cases, Text document analysis

## Abstract

This research aimed to provide a new decision pattern toward the ultimate goal of improving Asian disaster management. The “water flow decision pattern,” which is likened to the natural flow of water, was proposed to facilitate smooth decision-making by decision makers. Text document analysis with emphasis on a qualitative technique was used as the major methodology. Five failure cases were analyzed: the sinking of the ferry Sewol in Korea, the drought in India, the SARS outbreak in China, the nuclear leakage in Fukushima, and the typhoon Haiyan in the Philippines. The key finding was that the water flow decision pattern comprehensively combines five decision factors, namely, weight, availability, timeliness, emplacement, and roundabout. Hence, Asian nations may consider its application as a theoretical frame in the future, after appropriate training and exercise are carried out.

## Introduction

Many nations in Asia, such as South Korea (hereinafter Korea), India, China, Japan, and the Philippines, have made various decisions during disaster response. This phase of disaster management and its duration are extremely difficult to predict. Accordingly, an increasing number of people have strongly called for an orderly system of Asian disaster response. To facilitate such a system, pattern recognition in related decision-making is considered as an urgent prerequisite for disaster response (Beven and Alcock 2012; Hernandez et al. 2022). A major research question therefore is: “What simple and robust decision pattern could be applicable for Asian disaster response?”.

The purpose of this research is to provide a new decision pattern for Asian disaster response toward the ultimate goal of reducing the human loss, economic damage, and psychological impact of disasters in Asia. The present work proposes the so-called water flow decision pattern by considering five failure cases: the sinking of the ferry Sewol in Korea in 2014, the drought in India in 2015–2016, the outbreak of severe acute respiratory syndrome (SARS) in China in 2002, the nuclear leakage in Fukushima in 2011, and the typhoon Haiyan in the Philippines in 2013. These five cases were selected because of their critical impact to the region, not only physically but also socially. In the water flow decision pattern, decision makers decide on certain disaster response actions by applying a process likened to the natural flow of water.

The water flow decision pattern is a new decision pattern for disaster response. It is the result of rigorous research; that is, the pattern was developed in reference to both the ecosystem and pragmatic experiences. Thus, it can be considered as neither a revised nor an improved pattern. The key finding of this study is that the water flow decision pattern allows decision makers to comprehensively consider five decision factors, namely, weight, availability, timeliness, emplacement, and roundabout. Accordingly, Asian countries may apply the pattern as a theoretical frame in the future, after the implementation of relevant training and exercise.

## Literature review

Decision-making involves selecting a logical alternative(s) from among diverse options through careful thought processing. This is an important step because any decision, whether good or bad, may have serious consequences to individuals, institutions, nations, or even the international community, which may potentially be affected by a disaster. Various researchers have discussed decision models in their specialization areas, such as management, engineering, mathematics, psychology, and international politics, among others (Rivera-Royero et al. 2016). Nonetheless, the issue of pattern recognition has thus far not been directly applied to the domain of decision-making, although a few researchers have recently started discussing its significance.

As shown in Table 1, three theories may be evaluated as major decision models, depending on the extent of assumed human rationality. First, human beings are rational, according to many economists. Many decision makers constantly optimize the expected outcome of their options during their decision-making process, as in the rational choice theory, the rational action theory, or the choice theory. Such decision makers recall their information based on their expectations, preferences, and beliefs. Vice versa, they believe that biased or suboptimal information is detrimental to their decision-making (Lightle 2016).

**Table 1:** Summary of major previous studies on decision-making

Units	Researcher/s	Contribution	Methodology applied	Study limitations
Complete rationality	William Jevons	Development of consumption choices to maximize happiness or utility	Statistics and econometrics	Predicted the outcome of choice without a related process
	Daniel Kahneman	Integration of the rational choice theory into psychology	Empirical studies	No certainty whether his theory is turned into collective actors’ strategic behavior
Complete irrationality	Michael Cohen, James March, and Johan Olsen	Creation of the garbage can model	Computer simulation	Use of computer simulation, which is not consistent with informal theory
	Henri Poincare, Edward Lorenz, James Yorke, Benoit Mandelbrot, and James Gleick	Formulation of the chaos theory	Mathematical formulae	Subjective method used to detect chaos
Bounded rationality	Herbert Simon	Proposal of the concept of bounded rationality	Survey method	Use of bounded rationality, which has a low degree of specificity
	Ariel Rubinstein	Modeling of bounded rationality	Modeling	Need for further research to convince economic theorists

Furthermore, although many decision makers decide or act in line with their individual preferences, their behaviors are generally rational. Similarly, various human behaviors under the scope of microeconomics have been well understood within the aspect of complete human rationality (Costa et al. 2019). In identifying a problem, evaluating multiple means, or implementing solutions, organized reasons and evidence are fully utilized. Based on these characteristics, the application of a rational decision-making model has been expanded to other fields, such as political science, sociology, philosophy, anthropology, warfare, and evolutionary theory, among others.

Second, human beings can be irrational at times, according to some decision models, including the garbage can model of Michael D. Cohen, James G. March, and Johan P. Olsen. Addressing ambiguous behaviors in 1972, these authors found that extremely aggregate uncertainty could cause irrational behavioral responses during the decision-making process. Various decision factors are completely mixed in a garbage can or in organized anarchies; hence, the decision makers have to choose in a disorderly manner. On the basis of complete irrationality, many decision makers decide without providing any solution and thus face the same problems. In short, the majority of decisions are made in oversight (Cohen et al. 1972). Similarly, the decision makers tend to punish their rivals and, thus, diminish their own rewards for as long as they continue to rely on their emotion.

To elaborate, when a certain group of decision makers choose their options under a garbage can model, the decision-making process is not rationally clear. Instead, organizational anarchy is actively at work during the decision-making. This means that multiple streams, such as negatively defined problems, politics, or other policy matters, collide with one another. Thus, the decision makers produce unintended outcomes (from their decisions), which are random, unpredictable, or even clumsy. In other words, a garbage can could be surrounded by not only problems, participants, opportunities, and solutions but also unending changes (Einsiedel Jr 1983). A number of researchers have agreed that this model disconnects decision makers, identified problems, and solutions by referring to unique terms, such as problematic preferences, fluid participation, and unclear technology, among others.

Third, according to Herbert A. Simon, human beings have bounded rationality in their decision-making (Simon 1967). Many individuals are rational at times but are irrational at other times. Thus, it is not so easy to predict how individuals will react to their environment. In other words, mainly because human beings have limited rationality, their decisions are not always rational or irrational. Rather, their decisions are based on an incremental, or bounded, rationality.

Similarly, Simon differentiated two kinds of humans, specifically, “economic” and “administrative humans.” The latter was initially proposed by Simon and later refined by James March and Richard Cyert (Gow 2008). Simon raised the applicability of administrative men to many individuals and organizations. To elaborate, “economic men” are entirely rational in their decision-making when they are under an environment of certainty. In contrast, “administrative men” have simplified and limited viewpoints on their problems due to a lack of information, ability, time, and resources, among others. The decision of administrative men is not absolutely rational, only satisfactory or adequate. On this point, Simon indicated the importance of carrying out training, having a standard operation procedure, and other similar measures in relation to human or organizational behaviors.

Regarding disaster response, many researchers have examined related decisions and have reported that the decision-making in this phase has unique characteristics. In particular, the decision on disaster response has been often related to either the presence or absence of a critical risk. A wrong decision could result in serious risks and, ultimately, destructive effects. These risks include human losses, economic damages, and psychological impacts. Considering that the decision-making in disaster response could mean either ensuring the safety of or risking one’s life, making relevant decisions has become of even higher importance to both disaster victims and decision makers (Wang et al. 2016).

Decision-making during the phase of disaster response has always been faced with by the unexpected. The majority of affected individuals have not expected such an emergency situation to happen to them. Similarly, many decision makers have found themselves in an unexpected situation. Further, a series of unanticipated problems or events have frequently arisen. Accordingly, unexpected needs have developed from the diverse aspects of decision-making.

It has thus become necessary for decision makers to adopt nontraditional decision tools, such as “adhocracy” (e.g., employee empowerment, risk-taking, individual initiative, and innovation), and flexible measures during disaster response. When traditional decision tools, such as a rigid hierarchy and inflexible methods are applied, stakeholders may not produce effective decisions. Because of the short response time or the rapidly changing environment, decision makers may prefer to apply nontraditional mechanisms, which include not only a nonhierarchical structure but also flexible ways of resolving problems (Kapucu and Garayev 2011).

In contrast, decision-making during normal times has relied on more formal, cooperative, and analytical procedures compared with decision-making during disaster response. That is, the latter has depended on more intuitive procedures than the former. Furthermore, decision-making during disaster response has often been less oriented toward an analytical process and, thus, has become a less consultative discourse (Bonn and Rundle-Thiele 2007).

During disaster response, each decision maker may view problems and alternatives differently. In particular, considering that a disaster is generally not a linear or straightforward occurrence but rather is devious and complex to understand, many decision makers naturally apply multiple criteria to help them efficiently decide on the issues. For example, decision makers may partner with each other on decisions about resource allocation by relying on several strategies, such as both qualitative and quantitative methods and varied technologies, among others (Vaisanen et al. 2016).

Some critics have pointed out that, despite their unique characteristics, decision theories on disaster response are not perfect, as many of these do not have clear decision frames (Nunen et al. 2016). Moreover, although several theories have frequently shown how to maximize utility, function, or measures in relation to disaster response, the decision factors have been imprecise in reality. In short, precise decision factors and decision frames should be included in the decision patterns.

To elaborate, some researchers have discussed multiple decision factors in the field of disaster response. There are a number of decision factors under a specific decision-making theory, given that disaster response is a complicated process and could also be ambiguous at times. In extreme cases, those factors are not only heterogeneous but also nonlinear, contextual, or contradictory. Researchers have identified various decision factors depending on their individual perspectives (Nooraie 2012). Among these, five decision factors have been heavily supported: (a) seriousness, insecurity, and perception; (b) human resources, resources management, and strategies; (c) right time, prime time, and timing; (d) location, place, and locality; and (e) external environment, changes, and chances.

In view of the above, the present work proposes a new decision pattern for the phase of disaster response. In summary, this research considers the diverse and unique features of decision theories or models in disaster response in relation to the proposed pattern, which incorporates not only a decision frame but also precise decision factors. Hence, its research value lies in the proposal and description of a new decision pattern to comprehensively cover the whole aspect of decision-making in disaster response.

## Research design

The method of text document analysis involves discovering meaningful text patterns for a specific purpose by analyzing documents and then extracting appropriate information from them. However, as long as researchers depend solely on text document analysis, they are bound to always reach the same conclusions. This is a negative outcome of automated machine learning. To the point, the method of text document analysis substantially needs a new way to classify and understand text documents. In particular, text document analysis requires a qualitative technique that is not machine-driven but rather human-centered (Andric et al. 2021). In short, human interpretation of text documents is urgently needed to enhance the metric-driven process of text document analysis.

Text document analysis with emphasis on a qualitative technique has two clear advantages or contributions: the externalization of implicit assumptions and a less difficult process for researchers (Sinkovics 2018). The methodology externalizes implicit mental assumptions to the fullest extent. Hence, many readers will find it easier to understand and retrace the theoretically evolving process. In addition, because the methodology includes precise operationalization, clear-cut theories, and profound contextualization, researchers can smoothly design and implement related research structures.

In view of the above, the present work used text document analysis, which involved choosing and then interpreting several text documents on the topic. Similarly, the interpretation of text documents entailed understanding the cases of disaster response while producing meaningful analyses. Fundamentally, this improved methodology required a certain depth of interpretation. Basic concepts were extracted from various text documents as appropriate contexts were delineated. These contexts were used to identify the conditions surrounding persons, interactions, problems, or situations in the field (Corbin and Strauss 2008).

Internet search engines, such as ScienceDirect, EBSCOhost, Google Scholar, and Yahoo, were used. The search keywords included “decision-making pattern,” “disaster response,” “disaster management,” “text document analysis,” “qualitative analysis,” and combinations of these words. The methodology aided the development of a new decision pattern in disaster response based on the review of the related literature.

**Fig. 1:**
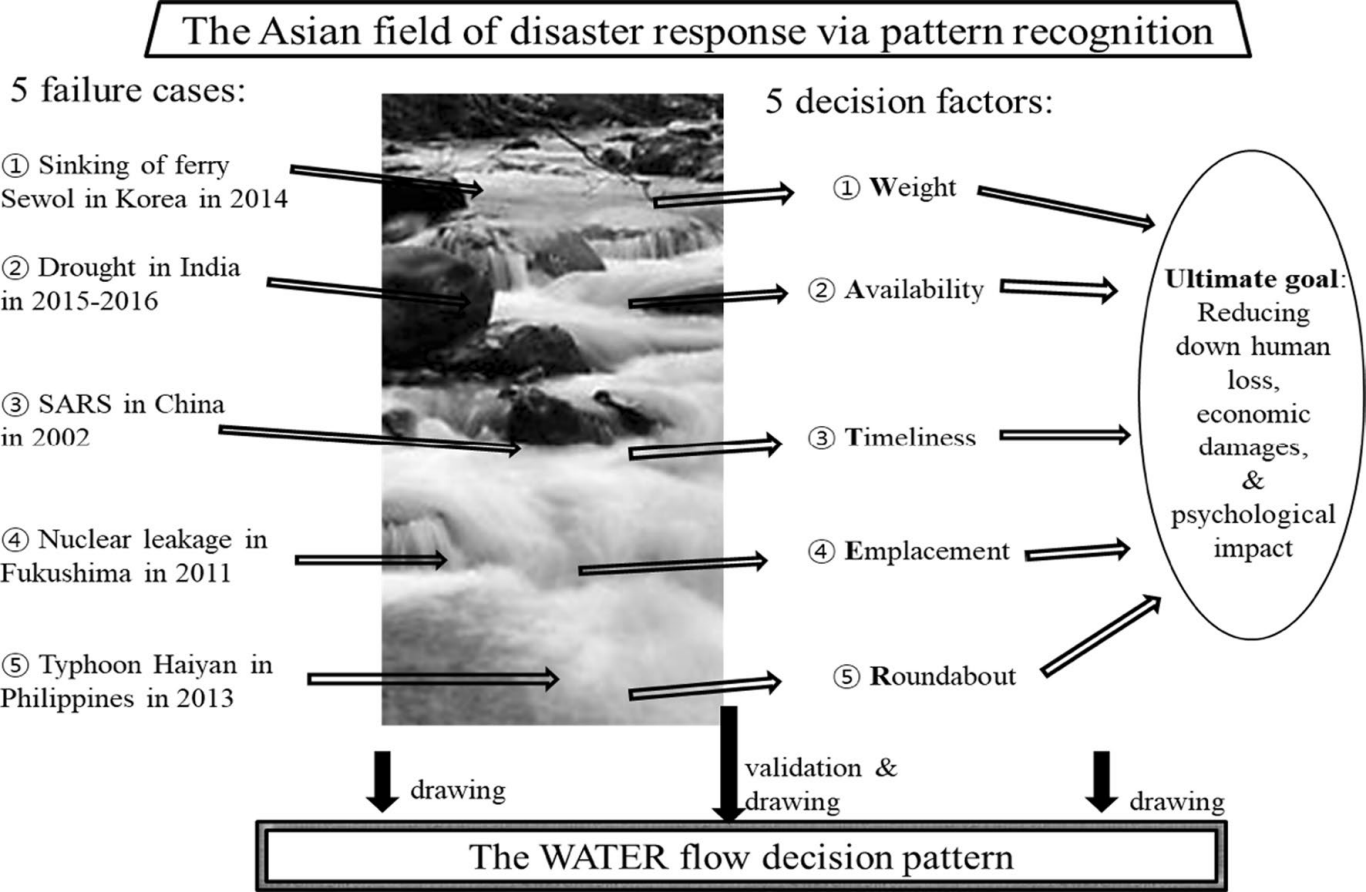
Water flow decision chart

The research did not cover the whole international community but rather was limited to Asian nations due to the specific research domain. On examination of various disaster responses within Asia, the water flow decision pattern was developed, in particular by carefully studying five failure cases in Asian disaster response and then identifying five decision factors (see Fig. 1).

Under the water flow decision pattern, the field of disaster response should make a decision(s) based on a process likened to the natural flow of water. When faced with numerous challenges in a complicated environment, the field needs to comprehensively consider all decision factors, as though carefully and strategically drawing objects from flowing water. Five decision factors were derived from diverse previous studies, although each prior work used a different name for each factor. Intentionally, the first letters of the five major decision factors form the acronym “WATER.” The five decision factors cover all significant aspects of disaster response in Asia, while maintaining a similar context with characteristics of a comprehensive nature.

The development of a new decision pattern may yield learn important lessons for the field of disaster response from either success or failure cases. In the present work, failure cases were considered because they are likely to provide more valuable information on disaster response compared with success cases. In other words, failure cases provide more deviant information than success cases. This allows the creation of a new decision pattern not by relying on consistent information but by embracing unexpected information (Labib and Read 2015).

Further, success cases can depreciate related information and knowledge on disasters more quickly compared with failure cases. Similarly, the magnitude of failure cases with the support of prior experience may affect how efficiently practitioners the field of disaster response learn important lessons. The greater the magnitude of failure, the more can be learned from it.

Given this rationale, this study included five cases into the category of failure in disaster response. Each case has been directly or indirectly evaluated as a failure case in the field based on the extensive literature review. Further, each case has had huge impacts in various local communities in Asia in terms of not only human loss and economic damage but also psychological impact. In fact, many Asians still remember the devastating effects of these disasters.

Therefore, it is necessary to validate how the five decision factors were drawn from the five failure cases selected. To achieve qualitative validity, the researcher(s) should determine or check whether the findings are accurate or inaccurate by applying multiple strategies (Creswell 2009). In this context, the author, as researcher, attempted to validate the five decision factors by using three strategies. First, a peer debriefer reviewed and then probed the relationship between the five decision factors and the five failure cases. Second, an external auditor reviewed the whole manuscript. Finally, the author applied descriptive analysis to deliver the findings. By describing the themes, settings, and other perspectives, the outcomes of this research are expected to be more realistic and, thus, validated.

## Five failure cases

### Sinking of the ferry Sewol in Korea in 2014

At approximately 8:50 a.m. on April 16, 2014, the ferry Sewol with 476 passengers onboard began to sink around Jindo, Korea. The sinking was reported to the emergency operation center in a neighboring maritime police station at 8:58 a.m. The headquarters of the Korea Coast Guard (currently known as the Ministry of the Interior and Safety) began to deploy a rescue team by 9:10 a.m., and the team arrived at the disaster area by 9:40 a.m. Only 172 of the passengers were rescued; many of those who died were high school students (MOIS 2022).

Various factors could explain why the ferry Sewol capsized, such as negligence on the part of the ferry captain, mismanagement by the ferry owner, and failure of the government response, among others. To elaborate, the captain of the ferry Sewol abandoned the ferry without first issuing an evacuation order to the passengers. He just tried to dry his wet paper money after running away from the ferry. The ferry owner illegally expanded the structure of his vessel to be able to carry more passengers and maximize his profits. The Korean president Park Geun-hye, who was then in the presidential palace Blue House, was later revealed to have taken no specific response to the crisis, since she was impeached.

However, the above factors share the fact that major stakeholders failed to recognize the weight or (potential) severity of the ferry Sewol sinking. If they had realized the gravity of the situation, they probably would have been more heavily involved in the related disaster response activities from the very beginning. It was unfortunate that even the captain and his crew abandoned the ferry without issuing an evacuation order. Simultaneously, the government missed a golden opportunity to show the nation how prepared it was in terms of providing efficient disaster response that could potentially save lives.

Regarding earlier theoretical models, such as complete rationality, complete irrationality, and bounded rationality, many factors around the sinking of the ferry Sewol were related to the theory of complete irrationality. That is, each factor, including negligence of the ferry captain, mismanagement by the ferry owner, and failure of government response, occurred under a high level of complete irrationality among decision makers.

### Drought in India in 2015–2016

One of the worst droughts in India was due to a heat wave that took place from 2015 to 2016, the impact of which was catastrophic. More than 330 million residents were affected by the drought; 2500 people lost their lives in 2015, and over 370 more died in 2016 (UNICEF 2016). In addition, because there was much less water than the yearly average in many reservoirs and basins, crop yields decreased significantly.

Climate change was determined to be the major cause of the drought. In particular, El Niño was blamed because it increased the temperature of the neighboring ocean. Similarly, many stakeholders were unable to appropriately respond to the long period of climate change in India. Some water suppliers failed to reduce the water supply in communities. Many local governments did not discuss, set up, or implement drought response plans in advance. Further, drought monitoring, early warning systems, and water efficiency programs, which should have been implemented in communities long before the occurrence of drought, were not in place.

However, a more fundamental cause of human loss was the lack of response resources given the above factors. The majority of those who died were residents of poor rural areas, who did not have air conditioners or sufficient ventilation in their homes. Further, these people did not have access to adequate medical facilities and drinking water. These response resources should have been a basic provision.

Moreover, in terms of the big picture, many factors around the drought in India were related to the theory of bounded rationality. The majority of Indians rationally recognized that they should neither contribute to nor facilitate climate change in the region; however, they realistically had no choice but to irrationally produce a large amount of contaminated air due to the matter of hunger or overcoming the poor economy.

### SARS outbreak in China in 2002

SARS is considered not only as a pneumonic disease but also as a zoonosis because the virus can be transferred from animals to humans through inhalation or close contact. SARS broke out in China in 2002, with the majority of cases reported in Guangdong and in Beijing. The disease spread over many other areas, including Shanxi, Shanghai, Sichuan, Hunan, Guangxi, and even Mongolia. About 5500 people contracted SARS, among whom 349 individuals died (ECDC 2021).

The traditional disaster response system of China failed to appropriately respond to the outbreak of SARS. For example, then Chinese president Hu Jintao did not seem to understand the issue of pandemic diseases or the related disaster management. He focused on firing incompetent officials in relation to SARS management efforts while relying on regimentation or exhortation. In short, he did not attempt to revolutionize the old system. Accordingly, many individuals and organizations had to accept the outbreak of SARS as their destiny or were left unaware of the extent of the impact of such a disaster.

That the Chinese government did not share information on the SARS outbreak with the public during the phase of initial response could be regarded as a major cause of human loss, considering that such nondisclosure by authorities was almost tantamount to a cover-up. Also, given that the global spread of SARS originated not from China but from a conference in Hong Kong, it seemed that Chinese authorities did not make solid efforts to disclose relevant information to the international community in a timely manner. Had timely actions been carried out, major human losses from SARS could have been decreased, if not prevented.

Among prior decision-making theories, the Chinese case of SARS outbreak was highly related to the pattern of complete rationality. From the traditional perspective, the Chinese population did not have to be made aware of the outbreak of SARS, which traditional thought even considered as a punishment from the gods. Hence, not undertaking any immediate disaster response to the outbreak was clearly rational at that time.

### Nuclear leakage in Fukushima in 2011

After the Tohoku earthquake in Japan, huge tsunamis hit the nuclear power plant in Fukushima on March 11, 2011. Nuclear reactors automatically shut down fission reactions. However, the tsunamis knocked down the cooling systems and then caused a series of explosions and meltdowns. The earthquake and resultant tsunamis killed almost 20,000 residents. Reportedly, the nuclear leakage in Fukushima did not directly cause any human deaths. However, neighboring areas were contaminated, leaving about 80,000 people at increased risk of cancer. These people may not be able to return to their homes for several decades due to the potential radiological exposure in the area (Demetriou 2011).

At first glance, some critical factors could explain why or how Japan failed to respond to the nuclear leakage in Fukushima. One of the major causes was related to the failure of the Tokyo Electric Power Company to meet the safety requirements for the nuclear power plant, such as risk assessment and preliminary damage assessment, among others. Another factor was that Japanese political leaders feared that the Japanese people would lose face if they sought international help to clean up their own nuclear leakage. Japanese leaders believed that Japan could handle its own disasters better than other countries could. Due to such an inferiority, or perhaps superiority, complex, Japan did not initially allow international teams to provide direct assistance in dealing with the Fukushima nuclear leakage.

Nonetheless, a fundamental cause of the nuclear leakage was that the nuclear power plant was located (or emplaced) in Fukushima, an earthquake-prone area within the Pacific Ring of Fire. Considering the long history of tsunami-generating earthquakes in Japan, a safer location should have been chosen for the nuclear power plant. Many former Japanese and current Japanese researchers have continuously warned of the danger of situating hazardous materials in local communities due to the potential for natural disasters in the region.

The Fukushima disaster was a typical case of limited rationality. The malfunction of the Tokyo Electric Power Company was quite irrational. The company should have claimed responsibility for related damages. However, the Japanese inferiority or superiority complex was rational, leading national leaders to follow their own psychological preferences. If they had been aware of their lack of professional knowledge, they would have sought international help from other advanced nations.

### Typhoon Haiyan in the Philippines in 2013

Haiyan, a category 5 typhoon, hit the Philippines, China, Taiwan, and Vietnam on November 8, 2013. Among these nations, the Philippines had the greatest number of human losses. More than 10,000 people died, and the catastrophe shocked the whole nation. At the same time, typhoon Haiyan caused huge economic damage to the nation, in addition to the physical devastation and destruction in many provinces and cities, such as Tacloban in Leyte (Thura 2013).

Similarly to any disaster case, multiple reasons could be cited to explain how a natural disaster came to devastate certain regions of the Philippines in 2013. For example, local governments did not allocate safe areas to accommodate the large number of evacuees as an initial response. The impact of Haiyan was particularly more severe among the poor than among the rich. Also, the nation failed to establish coordination efforts among various individuals and organizations within the short period of disaster response.

Although typhoon Haiyan was a cataclysmic phenomenon, its impact could have been lessened if the actions taken had not been roundabout or circuitous (In other words, the actions were not directly taken due to visible and invisible barriers) or, simply, if corruption had not been rampant in government. Later assessment showed that compliance to building codes was not appropriately observed or enforced in several structures in the affected areas. Additionally, local government and relief funds were gravely misused.

Considering the failure of local governments to allocate safe evacuation areas, the unfair treatment of the poor, and the lack of coordination, this case in the Philippines seemed to be a model of complete irrationality. However, these three factors were related to other elements in society, such as politics, economy, and culture. In this context, the case of Haiyan should be in line with complete rationality under a larger frame.

## Five decision factors

### Weight

The weight of a specific disaster affects all aspects of the related risks and disaster response. When the weight is light, the disaster is not likely to cause major damages. However, when the weight is heavy, the disaster presents serious risks for local communities, such as human loss, economic damage, and psychological impact. Thus, the term “weight” is equated to the disaster severity, seriousness, and depth, or the extent of its impact (Zhang et al. 2014).

Failure to accurately assess the weight of a disaster could result in many unresolved challenges, as in the case of the ferry Sewol sinking, in which the disaster response was miscalculated and miscarried, causing the loss of young lives and a severe emotional breakdown in the community and even in the whole nation. The correct identification of the weight of a specific disaster is critical, as is an aggressive response to the disaster. Otherwise, the related risks would be heightened or would become more complicated. Therefore, depending on the preliminary damage assessment, decision makers need to be systematic and decisive in determining the specific and appropriate disaster response actions.

### Availability

To effectively respond to disasters, a core team or a local institution should be available within every area that could be potentially affected. The team should include medical staff as first responders, as should have been the case during the heat stroke caused by the drought in India in 2015–2016. Firefighters could play a similar role to medical staff in responding to drought. Moreover, professional emergency managers could carry out appropriate actions in response to various emergencies (Jackson et al. 2002).

Similarly, material resources should be available in local communities. Although developing nations may not always have sufficient economic resources, they must prepare appropriate resources to prevent worst-case scenarios. The impacts of a disaster are likely to double when there is a failure to provide material resources, such as drinking water, sandbags, fire extinguishers, communication equipment, and personal protective equipment. By locating, allocating, or routing material resources, practitioners in the field of disaster management, particularly disaster response, may be able to respond and take action even when multiple disasters occur at the same time.

### Timeliness

The timing of the disaster response is extremely critical: this is the interval between the point when the decision makers respond and decide and the point when affected residents follow their advice and take action for their own safety. As soon as an emergency warning or advisory is communicated, residents should act accordingly and swiftly in seeking safe locations. Otherwise, they may end up as casualties or victims, as in the case of the SARS outbreak in China (Lovreglio et al. 2015).

In particular, decision-making during the phase of disaster response must take place in real time because many disasters take place without warning. To elaborate, when the period of disaster response is over, the related decision-making may no longer be useful at all. As a result, the related risks could gravely affect the whole community. Therefore, before the period of disaster response is over, the related decision-making should have been made and then implemented in real time.

### Emplacement

Planners in the field of disaster response should always consider where to emplace critical infrastructure, such as nuclear power plants; bridges, airports, and ports; and munition factories, to minimize, if not eliminate, concerns regarding the impact on disaster-prone locations. It is also important for decision makers to determine where to emplace the incident command post, emergency distribution centers, emergency shelters, and other critical facilities during disaster response (Paul and MacDonald 2016).

Additionally, whenever possible, the exact location of a disaster should be immediately known and circulated during the phase of disaster response. Without such knowledge, decision makers may find it difficult to take significantly aggressive actions quickly. For example, when an airplane is reported missing in an open area, the decision makers will find it difficult to make a definitive decision because they do not have timely knowledge of the exact location of the aircraft or its black box. Thus, they may not be able to progress to the next level of decision-making at the crucial time.

### Roundabout

The field of disaster response is so complicated that decision makers have been frequently faced with unexpected obstacles, as in the case of the typhoon Haiyan in the Philippines in 2013. In particular, not only visible but also invisible roundabouts are likely to have a negative impact during the period of disaster response. For examples, the lack of transparency during crisis communication is a good case of roundabouts, because it has not facilitated the public’s trust. Government institutions without appropriate accountability is another case of roundabouts, as analyzing that some of them have not directly disseminated warning messages to multiple residents (Dekongmen et al. 2021). Simultaneously, divisive politics also affects decision-making. For this reason, some decision makers have experienced high levels of emotional stress.

Another challenge to disaster response or related decision-making is the uncertainty involved. The extent of uncertainty increases through associated roundabouts when decision-making procedures, the interrelationships among them, or complicated issues are not clearly defined (Ghavami et al. 2019). Therefore, decision makers should systematically consider the ever-changing roundabouts in the scope of their decision-making.

## Implications of the water flow decision pattern

### Implication in practice

Personality and culture play a role in disaster response. Thus, individual differences are reflected in the decision-making during disaster response and thus are potentially embedded into the water flow decision pattern. This pattern contributes toward providing Asian countries with a decision frame by bringing together various disaster management principles and crucial factors.

The water flow decision pattern is a kind of multiple criteria model because it includes not only one but five decision factors in Asian disaster response. In the reality of disaster response, conflicting criteria interact with one another (Lu et al. 2016). Similarly, the water flow decision pattern comprehensively includes all five criteria under a complicated environment and then chooses the best option for disaster response.

The water flow decision pattern reflects the importance of simultaneous operation. In particular, it observes or supports simultaneous operation or execution because all five factors are equally considered during the decision-making process. If any one of the five decision factors, especially timeliness, were missing, the decision pattern would not be successful in providing the best option for disaster response.

The water flow decision pattern was drawn from practical experiences or pragmatic procedures in the field of disaster response, as shown in the five real-life cases. Similarly, whereas the flow of water is taken as the decision frame, the five decision factors may be considered as precise decision factors. Although the pattern is a theoretical frame, its feature is less philosophical and its application is ethical compared with other decision models. The process of pattern extraction shows that the decision pattern is a practice-based approach.

The water flow decision pattern is clearly simple in terms of its structure, being patterned after the natural water flow in the ecosystem. Considering that most individuals recognize the operational mechanism of the flow of water in a stream, the decision pattern is fairly easy to understand and, given its simple structure, may be applied as a fundamental theory by decision makers during disaster response.

### Social implication

To substantially apply the water flow decision pattern, decision makers have to share their own information or decision with each other. By sharing in the common goal of decision-making, all decision makers can contribute to the direction of the disaster response. Similarly, all decision makers must share their decision-making with the community as well. Otherwise, without the understanding of the residents, any decision arrived at would be entirely useless to the affected community (Rose et al. 2019).

In the Asian field of disaster response, each decision maker is surrounded by bounded rationality, which is also applicable to the water flow decision pattern. To overcome limited rationality or to better implement the water flow pattern, all decision makers need to apply reason or sound reasoning to their decision-making process. This entails the application of logic, calculation, or other problem-solving mechanisms, including collecting valid evidence.

However, the water flow decision pattern is not based solely on incremental decision, which is a typical case of bounded rationality. Instead, it is based partially on a dynamically acute decision-making process. Because decision-making within a given time period is subject to unexpected, complicated, and ever-changing factors, the decision pattern should be implemented with urgency. Further, the decision makers would have to make totally new decision(s) frequently. Therefore, a dynamically acute decision capability is one aspect of the water flow decision pattern.

### Managerial implication

In general, many decision theories have potential limitations in their application, which also restricts the scope of related research. Disaster response models, including the water flow decision pattern, are expected to contribute to the efficiency of disaster response, which focuses on effective operations. The water flow decision pattern has potential efficiency because it may be applicable to any disaster case, whether on land, ocean, or air. This makes it better than other decision models from the perspective of disaster response efficiency.

As concrete evidence, a group of previous disaster response theories in 2010 was applied to various isolated cases. Each theory was able to explain a certain aspect of disaster response, such as risk perception, resiliency, capacity building, and vulnerability assessment, among others (Patterson et al. 2010). In contrast, the water flow decision pattern may be applicable to any aspect of disaster response, in particular as a fundamental frame and within a short period of time. Consequently, it can produce better outcomes for disaster response efforts.

Nevertheless, the water flow decision pattern should be implemented not only once in relation to a specific disaster but rather should be applied repeatedly. Because the characteristics of disasters change rapidly, decision makers should continue to collaborate as they apply the water flow decision pattern, whereas stakeholders need to be decisive and at the same time flexible in implementing it. Otherwise, decision makers could end up in a cycle in which they are “chased” by one disaster after another.

At the same time, the water flow decision pattern itself should be regularly evaluated for sustainability. Therefore, it is necessary for practitioners in the field of disaster response to assess how the five factors are addressed for a specific disaster. By continuously balancing the five factors, the water flow decision pattern would be positively sustained.

Further, the field of disaster response needs to incorporate the water flow decision pattern into disaster management training and exercise. By learning or applying the process of the decision pattern during a simulated emergency situation, stakeholders would become aware of its importance and would be equipped to carry it out during the phase of disaster response in the future.

During training and exercise, certain scenarios should be simulated to maximize opportunities for learning. In addition, some exercises would have to be done repeatedly to achieve familiarity and mastery in preparation for real-life occurrences. Such exercises also foster calm and quick thinking under pressure as opposed to being panicky.

## Conclusion

This research aimed to develop a new decision pattern in Asian disaster response and thus contribute to achieving the ultimate goal of disaster management. The water flow decision pattern was introduced and described by analyzing five failure cases, namely, the sinking of the ferry Sewol in Korea, the drought in India, the SARS outbreak in China, the nuclear leakage in Fukushima, and the typhoon Haiyan in the Philippines.

The key finding of this work is that the water flow decision pattern comprehensively includes five decision factors, namely, weight, availability, timeliness, emplacement, and roundabout. The natural flow of water is taken as the decision frame, whereas the five decision factors are considered as precise decision factors. Therefore, Asian nations may flexibly consider the five factors and the flow of water in addressing and resolving disaster response challenges.

To effectively apply the water flow decision pattern, the stakeholders in the field of disaster management, particularly disaster response, should be given relevant training and exercise from the long-term perspective. This emergency training and exercise should include not only discussion-oriented activities, such as orientation, tabletop exercises, seminars, and games, but also practice-oriented tasks, such as drills, functional exercises, and full-scale exercises. In this way, the future decisions of stakeholders will be based on or guided by the water flow decision pattern.

Researchers who appreciate the strength of this research could extend future studies on the water flow decision pattern by elaborating on the five decision factors in terms of how specific nations have applied them in their own disaster management activities, particularly disaster response. Some researchers may also find it worthwhile to do comparative studies between this decision pattern and other theories applied to disaster response. Vice versa, researchers who disagree with the strength of this work could develop their own decision patterns in contrast to the water flow decision pattern. In sum, future collective efforts are expected to further strengthen the overall disaster response in Asia.
